# Budget impact of secondary hyperparathyroidism treatment in chronic kidney disease in an Ecuadorian social security hospital

**DOI:** 10.1186/s12913-016-1671-4

**Published:** 2016-08-26

**Authors:** Luis Manjarres, Pilar Sanchez, María C. Cabezas, Marco Fornasini, Valeria Freire, Adelin Albert

**Affiliations:** 1 Nephrology Service, Carlos Andrade Marin Hospital, Quito, Ecuador; 2 Medical School, Pontifical Catholic University of Ecuador, Quito, Ecuador; 3 Translational Research Center, Universidad de las Américas (UDLA), Quito, Ecuador; 4 Health & Research Services, Quito, Ecuador; 5 Public Health Department, University of Liège, Liège, Belgium

**Keywords:** Renal kidney disease, Calcitriol, Paricalcitol

## Abstract

**Background:**

Chronic kidney disease (CKD) is a disorder with high morbidity and mortality worldwide whose complications generate multiple costs. In Ecuador, only a few healthcare institutions have implemented management protocols aimed to reduce costs and to improve the quality of life of patients. The aim of this study is to evaluate the short-term (1-year) and long-term (5-year) costs and savings in the management of secondary hyperparathyroidism (SHPT) of hemodialyzed CKD patients by comparing calcitriol and paricalcitol in a large social security hospital in Quito, Ecuador.

**Methods:**

The estimation model assessed the resources used in the management of SHPT by comparing prospectively the cost savings within 1-year and 5-year time horizon with calcitriol and paricalcitol. Hospitalization, erythropoietin (EPO), treatment doses, intravenous iron consumption, and medical supplies were estimated according international references, based on the initial parathormone level (iPTH) of patients. The Ecuadorian National Reference costs (2014–2015) and institutional costs were used to calculate treatment costs. A statistical sensitivity analysis was also performed.

**Results:**

The study was based on data from 354 patients of whom 147 (41.4 %) had a value of iPTH in the range 300–600 pg/ml, 45 (12.8 %) in the range 601–800 pg/ml, and 162 (45.7 %) over 800 pg/ml. The 1-year estimated costs per patient for calcitriol and paricalcitol, respectively, were: medication, 63.88 USD and 1,123.44 USD; EPO, 19,522.95 USD and 16,478 USD; intravenous iron 143.21 USD and 187.76 USD. Yearly hospitalization costs per patient were 11,647.99 USD with calcitriol and 8,019.41 USD with paricalcitol. Total yearly costs per patient amounted to 31,378.02 USD with calcitriol and 25,809.50 USD with paricalcitol. Total savings using paricalcitol were 5,568.52 USD per patient compared with calcitriol. The 5-year cumulative medication costs were 319 USD for calcitriol and 2,403 USD for paricalcitol; EPO with calcitriol was 97,615 USD and with paricalcitol 82,394 USD; intravenous iron with calcitriol was 716 USD and paricalcitol 939 USD. Hospitalization costs for patients with calcitriol and paricalcitol were 43,095 USD and 62,595 USD, respectively. Total savings using paricalcitol amounted 32,414 USD per patient compared with calcitriol.

**Conclusions:**

Paricalcitol use generated more cost savings than calcitriol after 1 and 5 years.

## Background

Chronic kidney disease (CKD) is a clinical condition, which affects approximately 10 % of the adult population with high morbidity and mortality worldwide [1, 2]. According to the Vivenkanad study in 2013, the overall prevalence of CKD ranged between 8 and 16 %. The same study reported that in Latin America, the prevalence increased by about 7 % during the last few years [3]. The increasing frequency of type II diabetes and hypertension have greatly contributed to increased prevalence of CKD [4, 5].

A common complication of CKD is secondary hyperparathyroidism (SHPT), which generates high treatment costs. SHPT increases the frequency of skeletal and cardiovascular disorders [6]. SHPT is also known to worsen renal-driven anemia because higher levels of circulating parathyroid hormone is associated with a lower response to recombinant human erythropoietin [7]. Furthermore, SHPT is associated with increased hospitalization rates, especially when parathormone (PTH) levels exceed 600 pg/ml [8]. Thus, SHPT generates high costs to healthcare systems [9].

An Argentinian study published in 2013 reported that among 1210 CKD hemodialyzed patients, 26.7 % had an initial parathyroid hormone (iPTH) > 300 pg/ml [3]. In Ecuador, the total number of SHTP cases is not known; however, several studies based on relatively small samples have exhibited differing prevalence levels. As an illustration, a study (88 patients) in Guayaquil, Ecuador reported 3.4 % of SPTH cases in dialysis patients in 2009 [10], while another study (2012), in Ambato, Ecuador, on 54 hemodialyzed patients with CKD reported 32 % of the population with values > 450 pg/ml of iPTH [11].

The cost of care for CKD is high, especially in end stage renal disease (ESRD). In England (2012), the cost of CKD in 2009–2010 was estimated at £1.44 to £1.45 billion [12, 13]. Patients with ESRD consumed important economic resources from healthcare systems, with an estimated annual cost of 41,341.05 USD [14, 15]. Moreover, costs of CKD treatment have been increasing steadily; for example in the United States, they grew by 57 % between 1999 and 2004 [16]. In Latin American countries, the costs range from 10,956 USD to 14,654 USD per year per patient [17].

Calcitriol has been considered to be the first choice drug for treating SHPT in ESRD [18]. It is effective and inexpensive, although its use is frequently limited for patients with either hypercalcemia or hypophosphatemia. Other drugs like paricalcitol have been developed to treat these conditions [18]. Unlike calcitriol, paricalcitol has minimal impact on serum calcium and phosphorus [9]. However, paricalcitol is much more expensive than calcitriol. In Colombia (2016), the price of one vial of paricalcitol (5 μg) is 79,400 Colombian pesos (26.46 USD), while 0.25 μg of calcitriol costs 5,018 Colombian pesos (1.67 USD), reflecting the higher cost of paricalcitol, according to the Colombian Ministry of Health [19]. In Ecuador (2016), the price of paricalcitol is greater than calcitriol according the price of one vial of paricalcitol is 31.25 USD, while 0.25 μg of calcitriol costs 0.21 USD according the Ecuadorian Social Security Institute [20].

The aim of this comparative study was to evaluate the budget impact associated with calcitriol and paricalcitol in the management of secondary hyperparathyroidism (SHPT) in hemodialyzed patients with chronic kidney disease (CKD) 1-year and 5-year time horizons.

## Methods

The budget impact model was developed in Microsoft Excel to calculate the costs of management of hemodialyzed patients hospitalized with SHPT due to chronic kidney disease in a social security hospital in Quito, Ecuador. The study included data of patients from the nephrology department with a diagnosis of SHPT, who were under dialysis treatment between September 2013 and October 2014. Eligible patients were identified using code E21.1 of the International Classification of Diseases (ICD-10) for SHPT.

The only inclusion criterion was that the patient had been under SHPT dialysis treatment with either paricalcitol or calcitriol. Data collection consisted of a retrospective chart review of the institutional database. Serum iPTH was recorded during the previous 12 months in patients with calcitriol and paricalcitol treatment.

The study developed a model to estimate the annual cost of paricalcitol and calcitriol treatment. The analysis used international studies published to calculate the costs of management and treatment. We implemented an estimation to calculate the impact budget over 1 year and 5-year periods.

### Literature review

**Fig. 1:**
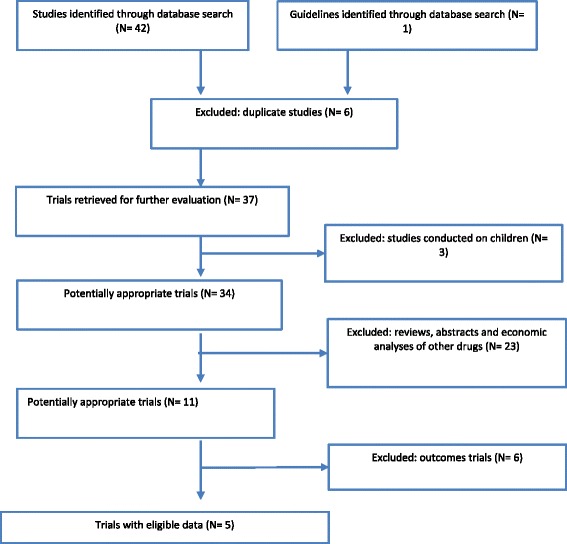
Flowchart for study selection

A systematic search was conducted in PubMed and Medline database in order to identify all randomized clinical trials of SHPT treatment, particularly those that compared paricalcitol and calcitriol treatments with respect to efficacy and which were conducted between January 2004 and December 2013. Trial selection criteria were based on study variables such as hospitalization days, iron, and erythropoietin consumption. Paricalcitol and calcitriol doses were determined from international effectiveness trials and guidelines (see Fig. 1).

The study of Naves Diaz was the reference to classify the initial PTH level (iPTH). The study classified patient iPTH level in three groups (group I: 300–600; group II: 601–800; group III: >800 pg/ml) in order to determine the amount of paricalcitol required for SHPT treatment [21]. Llach study provided the paricalcitol dose reference for group I. For groups II and III, doses were calculated based on a simple linear extrapolation [22]. The National Kidney Foundation Kidney Disease Outcomes Quality Initiative (NKF KDOQI) guidelines were the reference to determine the amount of calcitriol needed to treat SHPT. The guidelines recommend 0.25 μg of calcitriol per day [23].

The Capuano study was used as the reference to determine erythropoietin and intravenous iron yearly costs. This study showed a significant difference in the use of erythropoietin when using paricalcitol versus calcitriol [24]. As shown in Table 1, there are statistically significant differences in the weekly consumption of erythropoietin and intravenous iron in patients treated with paricalcitol and calcitriol. The calculations in the study used the annual consumption for each therapy (Table 1).

**Table 1:** Annual consumption of erythropoietin and intravenous iron according Paricalcitol and Calcitriol therapies

Parameter	Paricalcitol	Calcitriol	*P*-value
Erythropoietin Weekly Average Consumption	11,758 UI	13,930 UI	<0.05
Intravenous Iron Weekly Average Consumption	59 mg	45 mg	<0.05

Source: Capuano, A., 2009

Hospitalization were calculated based on the Dobrez study based on a sample of 11,443 patients, of whom 4,611 received treatment with paricalcitol and 6,832 with calcitriol [25]. The model used the averages of patients’ hospitalized rates and hospitalization days for each SHPT therapy (See Table 2).

**Table 2:** Hospitalization rates in patients with Paricalcitol and Calcitriol

Parameter	Paricalcitol	Calcitriol
Hospitalized patients per year per each 100 patients in treatment	59.6	75.2
Average number of hospitalization days per year	17.2	19.8

Source: Dobrez, DG., 2004

### Cost evaluation

The cost estimation included the resources used in the management of SHPT, such as erythropoietin and intravenous iron. Other variables considered in the model were erythropoietin consumption, intravenous iron consumption, hospitalization days, and medical supplies costs that were calculated based on international references [21–25]. Treatments costs were derived from “The Ecuadorian National Reference Costs”, published in December 2014 (see Table 3). Inflation was estimated from International Monetary Fund data.

**Table 3:** Unit cost according the National Reference

Category	USD
Paricalcitol 1 μg	1.25
Calcitriol 1 μg	0.84
Hospitalization Day	116.76
Erythropoietin 1 UI	0.03
Iron 1 mg	0.06

The paricalcitol price was established using the databases from the institution. The study calculated the cost of 1 μg for use in the model.

To calculate the annual cost of paricalcitol for each iPTH group, the model used the following formula:

$$ \mathrm{Paricalcitol}\ \mathrm{annual}\ \mathrm{cost}\ \mathrm{per}\ \mathrm{P}\mathrm{T}\mathrm{H}\ \mathrm{group}=\left[\begin{array}{c}\mathrm{P}\mathrm{aricalcitol}\\ {}\mathrm{micrograms}\\ {}\mathrm{required}\ \mathrm{per}\ \mathrm{year}\ \mathrm{per}\\ {}\mathrm{P}\mathrm{T}\mathrm{H}\ \mathrm{group}\end{array}\right] \times \left[\begin{array}{c}\mathrm{P}\mathrm{aricalcitol}\\ {}\mathrm{micrograms}\;\mathrm{price}\end{array}\right] $$

The average paricalcitol cost per year was used to estimate the cost per patient treatment using the following formula:

$$ \mathrm{Paricalcitol}\ \mathrm{cost}\ \mathrm{per}\ \mathrm{patient} = \Sigma \left[\begin{array}{c}\mathrm{P}\mathrm{aricalcitol}\ \mathrm{annual}\ \\ {}\mathrm{cost}\mathrm{s}\kern0.5em \mathrm{per}\kern0.5em \mathrm{P}\mathrm{T}\mathrm{H}\ \mathrm{group}\end{array}\right]\times \left[\begin{array}{c}\%\ \mathrm{patient}\mathrm{s}\\ {}\mathrm{per}\kern0.5em \mathrm{P}\mathrm{T}\mathrm{H}\\ {}\mathrm{group}\end{array}\right] $$

For calcitriol, the model considered the different presentations and the institutional price to estimate the drug cost per microgram.

Annual costs of calcitriol treatment per patient in the model were calculated using the following formula:

$$ \mathrm{Calcitriol}\ \mathrm{annual}\ \mathrm{cost}\ \mathrm{per}\ \mathrm{patient} = \left[\begin{array}{c}\mathrm{Calciotriol}\ \mathrm{dose}\ \\ {}0,25\ \mu g\end{array}\right]\times \left[\begin{array}{c}\mu g\ \mathrm{Calcitriol}\\ {}\mathrm{Price}\end{array}\right] \times \left[\begin{array}{l}365\\ {}\mathrm{days}\end{array}\right] $$

Erythropoietin was calculated based on 1 International Unit (IU) of cost according the institutional cost of erythropoietin. The annual cost of erythropoietin was calculated by multiplying the mean unit cost by the weekly consumption of erythropoietin and then multiplying by 52 weeks.

The cost of annual intravenous iron was calculated based on its institutional cost multiplied by weekly consumption and then multiplied by 52 weeks.

Hospitalization cost per patient was based on the price of 1 day of hospitalization according the “Ecuadorian National Reference Costs” including estimate the annual inflation.

Calculations were based on a simple linear extrapolation according the iPTH level and the literature review. Subjects with missing data were not included in the analysis.

A one-way sensitivity analysis was conducted by varying the following parameters by ± 10 %, hospitalization costs per day, average erythropoietin costs, average intravenous iron costs, treatment iPTH, hospitalization rate reduction, annual hospitalization reduction, erythropoietin weekly savings costs of paricalcitol and calcitriol, and the number of hemodialysis per month; an inflation rate by ± 2.5 % was included.

## Results

The study was based on data from 354 SHPT patients whose iPTH levels were registered. The average estimates presented in Table 4 were calculated according to the Naves-Diaz study, divided into three iPTH l groups achieving, respectively, 435 pg/ml in the first group, 668 pg/ml in the second group, and 1236 pg/ml in the third group.

**Table 4:** Initial PTH levels (*n* = 354 patients)

	Basal PTH group^a^	No. of patients	PTH mean level (pg/ml)
PTH serum levels	300-600 pg/ml	118	435
	601-800 pg/ml	67	668
	>800 pg/ml	169	1, 236

^a^Basal PTH ranges were according Naves-Diaz Study [21]

### Secondary hyperthyroidism costs

The projected cost per patient to treat SHPT using paricalcitol across time was 1,123.44 USD the first year and 2,403 USD the fifth year. The cost for calcitriol treatment was 63.88 USD the first year, and 319 USD the fifth year, as shown in Table 5.

**Table 5:** Cumulative costs per patient short and long term in USD

	1st Year		5th Year	
	Paricalcitol	Calcitriol	Paricalcitol	Calcitriol
	(A)	(B)	(A)	(B)
SHPT treatment	1,123.44	63.88	2,403	319
Erythropoietin	16,478	19,522.95	82,394	97,615
Intravenous iron	187.76	143.21	939	716
Hospitalization	8,019.41	11,647.99	43,095	62,595
Total	25,809.50	31,378.02	128,831	161,245

**Fig. 2:**
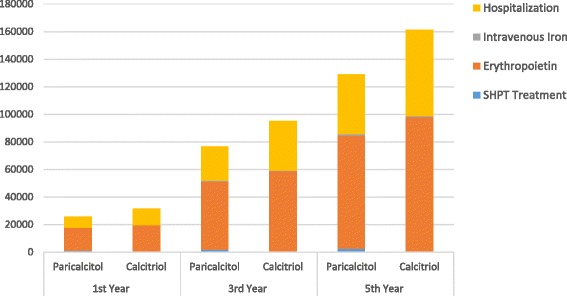
Cumulative savings per patient short and long term in US dollars

Hence, treatment with paricalcitol represented costs reductions of 5,568.52 USD the first year and 32,414.00 USD the fifth year (Fig. 2).

### Sensitivity analyses

The results of the one-way sensitivity analyses are shown in Table 6. The present model yielded a decrease around 18 % of costs in all parameters. Sensitivity analysis shown that the average treatment of SHPT with paricalcitol was less expensive than that of calcitriol.

**Table 6:** Cumulative Net Savings in 5 years

		5-year savings	
Parameter	Minimum savings (USD)	Maximum savings (USD)	Initial value (USD)
Daily hospitalization costs (±10 %)	52,427	80,178	66,303
Erythropoietin costs (±10 %)	61,389	72,683	66,303
Intravenous iron costs (±10 %)	66,214	66,391	66,303
Annual inflation 2014–2016 (±2.5 %)	56,397	76,876	66,303
Treatment costs per iPTH group (I: 29 %, II: 17 %, III: 42 %)	66,303	89,994	66,303
Hemodialysis per month (±10 %)	79.66	52,983	66,303
Weekly savings erythropoietin administration (±10 %)	60,164	72,435	66,303
Hospitalization rate reduction (±10 %)	58,276	74,329	66,303
Annual hospitalization reduction (±10 %)	61,667	70,938	66,303

## Discussion

This study shows that paricalcitol was the best economic option to treat patients with SHPT, saving 2,679.31 USD in 1 year and 16,249 USD in 5 years per patient treated. Treatment costs with paricalcitol were 18,799 USD for the short term (1-year) and 91,744 USD for the long term (5-year). Despite its higher costs compared with calcitriol, paricalcitol showed higher effectiveness and benefits. Patients treated with paricalcitol presented fewer hospitalizations (59.6 %), generating important savings in direct costs in the management of patients with SHPT.

According to the study of Nuijten et al. (2009) conducted in the United States, treatment with paricalcitol represented annual savings of 1,941 USD compared to calcitriol [26]. This results of that study are similar to those presented here, where paricalcitol savings per patient/year were 2,679.31 USD in the short term (1-year). The study published by Lorenzoni et al. (2014) demonstrated that paricalcitol used during the pre-dialysis stage and at early stage of SHPT in Italy represented an overall reduction in direct medical costs from 1′782,921 to 1′622,357 Euro for the healthcare system. The savings in a hypothetical cohort of 1,000 patients would amount to 1′971,500 Euro, which is less than the savings per 1,000 patients of 16′249,000 USD calculated in our model [27]. However, Lorenzoni study was implemented in pre-dialysis stage.

Another study, conducted in Mexico by Sanchez-Casillas et al. (2013), reported a total long term (5-year) cost of 24,532.88 USD using paricalcitol therapy compared to 35,633.36 USD using calcitriol therapy, which reflects greater costs for both therapies compared to those calculated in our study (91,744 USD vs 107,933 USD) [28]. Nevertheless, the Mexican study also found cost savings of paricalcitol compared with calcitriol in SHPT.

The work of Sprague et al. (2003) in the United States demonstrated that paricalcitol is more effective over PTH levels in SHPT patients, achieving a reduction of iPTH levels over 50 % after 18 weeks of treatment with less sustained hypercalcemia [29]. The control of serum calcium in SHPT patients is important for avoiding cardiovascular complications and parathyroid hyperplasia, thus leading to fewer hospitalizations, as described in the 2006 Cheng et al. review [30]. A study by Rosery et al. (2006) conducted in 6376 patients indicated that paricalcitol treatment resulted in a reduction of 84 % of hospitalizations in 1 year compared to calcitriol treatment, producing savings of 5,394 USD associated with paricalcitol treatment [31]. In sum, these studies demonstrated the cost savings of paricalcitol consistent with the present study.

In our model, erythropoietin was the most expensive medication used in the management of SHPT patients. Capuano et al. described the erythropoietin international units recommended for patients treated with paricalcitol compared with calcitriol. They showed that there is evidence of a need of lower amount of erythropoietin with paricalcitol therapy [24]. Afsar et al. and Riccio et al. (2015) showed that paricalcitol increased hemoglobin levels, decreased urinary protein excretion, and generated lower resistance to erythropoietin treatment; in addition, it did not interfere with erythropoietin synthesis [32, 33].

The present study has some limitations, most importantly, that the study population was limited to the main social security general hospital in Quito, Ecuador. Another limitation is that the study was not able to compare the cost of paricalcitol with calcinanet, which is not available in the Ecuadorian market. In addition, our model did not take into account the CKD stage of disease only the iPTH. Finally, the model was developed using economic simulations based on international parameters, which might not be applicable in Ecuador.

## Conclusions

In conclusion, the average annual cost per patient for paricalcitol therapy was 18,799, USD of which 80 % represented hospitalization and erythropoietin consumption. The use of paricalcitol, according to PTH initial level, represented substantial economic savings of 2,679 USD in the first year and 16,249 USD in the fifth year, when compared to calcitriol. The present study supports the use of paricalcitol as the first choice drug to treat SHPT in patients with chronic renal insufficiency.

## Abbreviations

CKD: Chronic kidney disease
EPO: Erythropoietin
ESRD: End stage renal disease
ICD-10: International classification of diseases
iPTH: Initial parathormone
IU: International Unit
NKF KDOQI: National kidney foundation kidney disease outcomes quality initiative (NKF KDOQI)
PTH: Parathormone
SHPT: Secondary hyperparathyroidism (SHPT)
